# Sunitinib in combination with trastuzumab for the treatment of advanced breast cancer: activity and safety results from a phase II study

**DOI:** 10.1186/1471-2407-14-166

**Published:** 2014-03-07

**Authors:** Thomas Bachelot, Jose A Garcia-Saenz, Sunil Verma, Maya Gutierrez, Xavier Pivot, Mark F Kozloff, Catherine Prady, Xin Huang, Reza Khosravan, Zhixiao Wang, Rossano Cesari, Vanessa Tassell, Kenneth A Kern, Jean-Yves Blay, Ana Lluch

**Affiliations:** 1 EORTC, Soft Tissue and Bone Sarcoma Group, Centre Léon-Bérard and Université Claude Bernard, Lyon, France; 2 Hospital Clinico San Carlos, Madrid, Spain; 3 Sunnybrook Health Sciences Centre, Odette Cancer Centre, Toronto, Canada; 4 Medical Oncology, René Huguenin Cancer Centre, Saint Cloud, France; 5 Hôpital Jean Minjoz, Besançon, France; 6 University of Chicago, Chicago, and Ingalls Memorial Hospital, Harvey, IL, USA; 7 Centre intégré de cancérologie de la Montérégie, CSSS Champlain-Charles-Lemoyne, Greenfield Park, Quebec, Canada; 8 Pfizer Oncology, La Jolla, CA, USA; 9 Pfizer Oncology, New York, NY, USA; 10 Pfizer Oncology, Milan, Italy; 11 INCLIVA-Servicio de Oncología Médica, Hospital Clínico Universitario de Valencia, Valencia, Spain; 12 Previous employee of Pfizer; current affiliation: Eisai Inc., Woodcliff Lake, NJ, USA; 13 Previous employee of Pfizer; current affiliation: Aragon Pharmaceuticals Inc., San Diego, CA, USA; 14 Léon Bérard Comprehensive Cancer Centre, Université Claude Bernard Lyon I, 28 rue Laennec, F-69008 Lyon, France

**Keywords:** Sunitinib, Trastuzumab, Advanced breast cancer

## Abstract

**Background:**

This phase II study evaluated the efficacy and safety/tolerability of sunitinib plus trastuzumab in patients with HER2-positive advanced breast cancer (ABC).

**Methods:**

Eligible patients received sunitinib 37.5 mg/day and trastuzumab administered either weekly (loading, 4 mg/kg; then weekly 2 mg/kg) or 3-weekly (loading, 8 mg/kg; then 3-weekly 6 mg/kg). Prior trastuzumab and/or lapatinib treatment were permitted. The primary endpoint was objective response rate (ORR).

**Results:**

Sixty patients were enrolled and evaluable for safety; 57 were evaluable for efficacy. The majority of patients (58%) had received no prior chemotherapy in the metastatic setting. The ORR was 37%; the clinical benefit rate (CBR; percent objective response plus stable disease ≥ 24 weeks) was 56%. Among patients who were treatment-naïve or had received only adjuvant therapy, the ORR was 44% and the CBR was 59%. Overall, median overall survival had not been reached and the 1-year survival rate was 91%. The majority of adverse events (AEs) were mild to moderate in severity. Forty percent of patients experienced AEs related to measured left ventricular ejection fraction (LVEF) declines, which occurred more frequently in patients who had received prior anthracycline treatment. Ten percent of patients exhibited symptoms related to LVEF declines. One patient died on study from cardiogenic shock. Antitumor response and several safety parameters appeared to correlate with sunitinib exposure.

**Conclusions:**

Sunitinib plus trastuzumab demonstrated antitumor activity in patients with HER2-positive ABC, particularly those who were treatment-naïve or had only received prior adjuvant treatment. Sunitinib plus trastuzumab had acceptable safety and tolerability in patients with HER2-positive ABC who had not received prior anthracycline therapy.

**Trial registration:**

NCT00243503

## Background

Trastuzumab is approved in combination with taxanes for first-line treatment and as monotherapy for second-line treatment of HER2-positive metastatic breast cancer (MBC). Objective response rates (ORRs) for patients with MBC receiving trastuzumab monotherapy were 26% in the first-line setting [1] and approximately 15% in the second-line setting [2,3]. However, resistance to trastuzumab alone or in combination with chemotherapy generally develops within 1 year of initiating treatment [1,2,4-6].

Sunitinib malate (SUTENT®; Pfizer Inc., New York, NY, USA), an oral, multitargeted tyrosine kinase inhibitor (TKI) of vascular endothelial growth factor receptors (VEGFRs), platelet-derived growth factor receptors (PDGFRs), and other receptor tyrosine kinases [7-12], is approved multinationally for the treatment of advanced renal cell carcinoma (RCC), imatinib-resistant/-intolerant gastrointestinal stromal tumor (GIST), and progressing metastatic pancreatic neuroendocrine tumor. In a phase II study of heavily pretreated patients with MBC (N = 64), single-agent sunitinib at 50 mg/day on Schedule 4/2 (4 weeks on treatment followed by 2 weeks off treatment) demonstrated antitumor activity (ORR, 11%; clinical benefit rate [CBR; percent objective responses plus stable disease (SD) ≥ 24 weeks], 16%) [13]. In some patients in this trial, tumor regrowth was observed during the period off treatment following initial shrinkage on treatment. Sunitinib administration at 37.5 mg on a continuous daily dosing (CDD) schedule has also been found to be active and feasible in RCC, GIST, and pancreatic neuroendocrine tumor [14-17], and was used subsequently in sunitinib trials in MBC.

Preclinical and clinical data support the rationale that concurrent inhibition of VEGF and HER2 signaling may be more efficacious than inhibition of either target alone [18-21]. VEGF may in part mediate the aggressive breast cancer (BC) phenotype associated with HER2 overexpression [18,19], and constitutively active HER2 has been shown to increase VEGF protein synthesis levels in human BC cells [20].

In a mouse model of HER2-amplified BC, sunitinib plus trastuzumab elicited a 75–80% greater decrease in tumor volume than either agent alone (Pfizer Inc., data on file). A phase II study of the anti-VEGF monoclonal antibody bevacizumab and trastuzumab as first-line therapy for HER2-positive MBC yielded an ORR of 46% [21], which compared favorably with results obtained with trastuzumab alone in other studies [1,22]. Preliminary results of a phase III study also suggested that addition of bevacizumab to the combination of trastuzumab and docetaxel in a similar setting leads to modest improvements in clinical outcomes [23]. Since preclinical studies had demonstrated that dual inhibition of the VEGF and PDGF signaling pathways provide greater antitumor activity than inhibition of either pathway alone [24,25], it was hypothesized that the addition of the multitargeted TKI sunitinib to a trastuzumab-based regimen would be especially efficacious.

The current phase II study evaluated the efficacy and safety/tolerability of sunitinib on a CDD schedule in combination with trastuzumab, weekly or every 3 weeks, in patients with HER2-positive advanced (metastatic or locally recurrent) BC (ABC). The primary objective of the study was to determine the antitumor activity of the combination. Sunitinib is not approved for ABC, and Pfizer did not submit a request for regulatory review of sunitinib in ABC by the FDA or other regulatory bodies. This decision was made following the findings that sunitinib did not meet primary study endpoints in phase III trials in this setting [26-29].

## Methods

### Patient selection

Key inclusion criteria: Eligible patients were female aged ≥ 18 years with histologically or cytologically proven, unresectable, locally recurrent or metastatic HER2-positive BC and measurable disease based on Response Evaluation Criteria in Solid Tumors (RECIST) [30]. The study also included patients who may have had prior trastuzumab and/or lapatinib treatment in the neoadjuvant, adjuvant, or metastatic disease setting, or prior treatment with hormone therapy in the adjuvant and/or advanced disease setting. Patients were required to have an Eastern Cooperative Oncology Group performance status of 0 or 1 with adequate organ function (including left ventricular ejection fraction [LVEF] ≥ 55%) and resolution of all acute toxic effects of prior therapy or surgical procedures to National Cancer Institute Common Terminology Criteria for Adverse Events, version 3.0 (NCI CTCAE v3.0) grade ≤ 1 (except alopecia).

Key exclusion criteria: Patients with prior treatment with more than one regimen of cytotoxic therapy for advanced disease or prior treatment with sunitinib (or trastuzumab if there was a history of hypersensitivity reactions) were excluded, as were patients with prior systemic therapy, radiation therapy, or surgery ≤ 3 weeks before the first dose of study treatment (prior palliative radiotherapy to non-target metastatic lesions was permitted). Brain metastases and cardiovascular disease or uncontrolled hypertension were also exclusion criteria.

This study was conducted in accordance with the International Conference on Harmonisation Good Clinical Practice guidelines, the Declaration of Helsinki, and applicable local regulatory requirements and laws. Approval from the institutional review board (IRB) or independent ethics committee (IEC) of each participating center was required. All patients gave written, informed consent prior to enrollment.

### Study design and dosing

Originally developed using a randomized, placebo-controlled design (control arm: trastuzumab plus placebo; test arm: trastuzumab plus sunitinib), the study was subsequently changed to an open-label, single-arm design in response to evolving standards of care in which single-agent trastuzumab was considered suboptimal for patient treatment. As such, with the control arm consisting of trastuzumab as the only active agent, patient recruitment was limited. Under these circumstances, the control arm was removed, while the test arm (trastuzumab plus sunitinib) was retained in the revised single-arm study. The primary endpoint was ORR based on RECIST. Secondary endpoints included duration of tumor response, CBR, progression-free survival (PFS), overall survival (OS), safety, pharmacokinetics (PK), and patient-reported outcomes (PROs). The final protocol was approved by the IRB and/or IEC at each participating center.

### Study-drug administration

Sunitinib 37.5 mg was taken orally once daily in the morning, without regard to meals; a cycle was considered to be 4 weeks. Patients were monitored for toxicity; 1-week dosing interruptions were permitted for dose-limiting toxicities. Dose reduction to 25 mg/day was permitted for recurring grade 3/4 toxicity. Dose escalation to 50 mg/day was permitted after two treatment cycles with minimal treatment-related side effects. Further dose titration was permitted in subsequent cycles based on tolerability.

Trastuzumab was administered intravenously starting on cycle 1, day 1 (C1D1), on either a weekly schedule (loading dose, 4 mg/kg on day 1; maintenance dose, 2 mg/kg on days 1, 8, 15, and 22 every 4 weeks) or a 3-weekly schedule (loading dose, 8 mg/kg on day 1; maintenance dose, 6 mg/kg every 3 weeks), as a previous study had shown no difference in efficacy or safety between the two schedules [22]. No dose modification was permitted, but dosing could be delayed depending on tolerability.

### Study assessments

Tumor assessments were performed using computed tomography or magnetic resonance imaging at baseline and every 8 weeks, and evaluated by the investigator using RECIST. Objective responses were confirmed ≥ 4 weeks after initial documentation.

Safety was evaluated at regular intervals by monitoring adverse events (AEs; NCI CTCAE v3.0), hematology, and blood chemistry, and by physical examinations. QTc intervals were monitored using triplicate 12-lead electrocardiograms. LVEF was assessed using multigated acquisition or echocardiogram scanning at screening, on day 1 of odd-numbered treatment cycles beginning with C3, as clinically indicated, and when treatment was discontinued.

Blood samples were collected pre-dose on C3D1 and C5D1 to evaluate trough concentrations of sunitinib and the active metabolite SU12662 using a validated, sensitive, and specific liquid chromatography-tandem mass spectrometric method (Bioanalytical Systems Inc; Lafayette, IN, USA) with acceptable accuracy and precision of quality control samples for sunitinib (0.7–1.7% and ≤ 6.6%, respectively) and SU12662 (-1.5% to 1.3% and ≤ 8.0%).

PROs were assessed using the self-administered European Organisation for Research and Treatment of Cancer Quality of Life Questionnaire C30 (EORTC QLQ-C30) and the related BC module BR23. EORTC QLQ-C30 assesses global health status, five functional domains (physical, role, cognitive, emotional, and social), eight symptom domains (fatigue, pain, nausea, appetite loss, constipation, diarrhea, dyspnea, and insomnia), and financial difficulties. BR23 assesses disease-related symptoms. Significant change was defined as both a clinically meaningful change of ≥ 10 points (minimum important difference) [31] and a 95% confidence interval (CI) for change from baseline not containing zero. Questionnaires were completed by patients on day 1 of each odd-numbered treatment cycle and at the end of treatment or withdrawal from the study.

### Statistical analysis

The historical trastuzumab ORR was assumed to be ≤ 20% [1-3] and the predicted ORR for sunitinib plus trastuzumab to be ≥ 33%. A sample size of ≥ 53 was required to have 80% power at a 10% significance level to detect a 13% improvement in ORR. The lower bound of the 95% CI of the ORR was required to be > 13% to reject the null hypothesis that the ORR of sunitinib plus trastuzumab was no different than that of the historical single-agent trastuzumab ORR.

The intention-to-treat population was the primary population for the evaluation of efficacy endpoints and patient characteristics. Exact two-sided 95% CIs for the ORR and CBR were calculated using a method based on the F distribution. Time-to-event endpoints were summarized using the Kaplan–Meier method. One-year survival probability was estimated using the Kaplan–Meier method, with a two-sided 95% CI calculated for the log using a normal approximation and then back-transformed to give a CI for the 1-year survival probability itself.

Steady-state dose-corrected trough plasma sunitinib concentrations were derived using data from patients who had taken sunitinib for ≥ 10 consecutive days by correcting observed concentrations in patients who underwent dosing modifications based on the 37.5-mg starting dose (starting dose ÷ actual dose).

PRO data analysis was limited to the first seven cycles, in which there were ≥ 10 patients.

## Results

### Patient characteristics

Sixty patients with median age of 54 years (range 31–81) and ECOG PS score of 0/1 in 52/58%, respectively, were enrolled in the study at 17 centers across five countries between May 2006 and July 2008: six on the original protocol and 54 under the amended open-label design. Patient demographics and baseline characteristics are shown in Table 1. There were 33 subjects (55%) who were estrogen receptor-positive and 22 subjects (37%) who were progesterone receptor positive. All subjects were HER2 positive by either FISH or IHC. The majority of patients (58%) had received no prior chemotherapy in the metastatic setting.

**Table 1:** Patient characteristics at baseline

**Characteristic**	**n (%)**
Patients receiving treatment	60 (100)
Median age (range), years	54 (31–81)
ECOG PS	
0	31 (52)
1	29 (48)
Disease stage	
Locally recurrent	2 (3)
Metastatic	58 (97)
Histologic type	
Ductal	51 (85)
Ductal and lobular	1 (2)
Ductal and other	1 (2)
Lobular	3 (5)
Other	4 (7)
Receptor status	
Estrogen receptor-positive	33 (55)
Progesterone receptor-positive	22 (37)
HER2-positive*	60 (100)
Prior systemic therapy	44 (73)
Anthracycline + trastuzumab or lapatinib	26 (43)
Anthracycline only	11 (18)
Trastuzumab only	4 (7)
Lapatinib only	2 (3)
Other	1 (2)
No prior systemic therapy	16 (27)
Prior chemotherapy in metastatic setting	
Yes	25 (42)
No	35 (58)
Location of disease	
Regional or distant lymph node	35 (58)
Lung	27 (45)
Bone	25 (42)
Liver	24 (40)

*Abbreviation*: *ECOG PS* Eastern Cooperative Oncology Group performance status.

*IHC 3+: 52 (87%); IHC 2+ 6 (10%); FISH+: 22 (37%).

### Treatment received

Fifty-seven patients received the sunitinib–trastuzumab combination, making them evaluable for efficacy. Three patients received only trastuzumab. All 60 patients were evaluable for safety/tolerability. By data cut-off (October 2010), two patients had completed the study after receiving 18 months of study treatment and the remaining 58 patients had discontinued treatment: 44 due to disease progression, 11 due to AEs, and one each to death, consent withdrawal, and other (unspecified) reasons. Median treatment duration was 3.9 months (range: 0.5–15.7). Among the 57 patients who received the sunitinib–trastuzumab combination, most (63%) had at least one sunitinib dosing interruption, with a median length of interruption of 8 days (range: 4–46). The sunitinib dose was reduced to 25 mg in 22 patients (39%) and subsequently to 12.5 mg in three patients (5%). Among the 60 patients who received trastuzumab, dosing was delayed by ≥ 1 week in 18 patients (30%). The median relative dose intensity was 72% (range: 47–127%) for sunitinib and 96% (range: 60–122%) for trastuzumab.

### Efficacy

**Figure 1:**
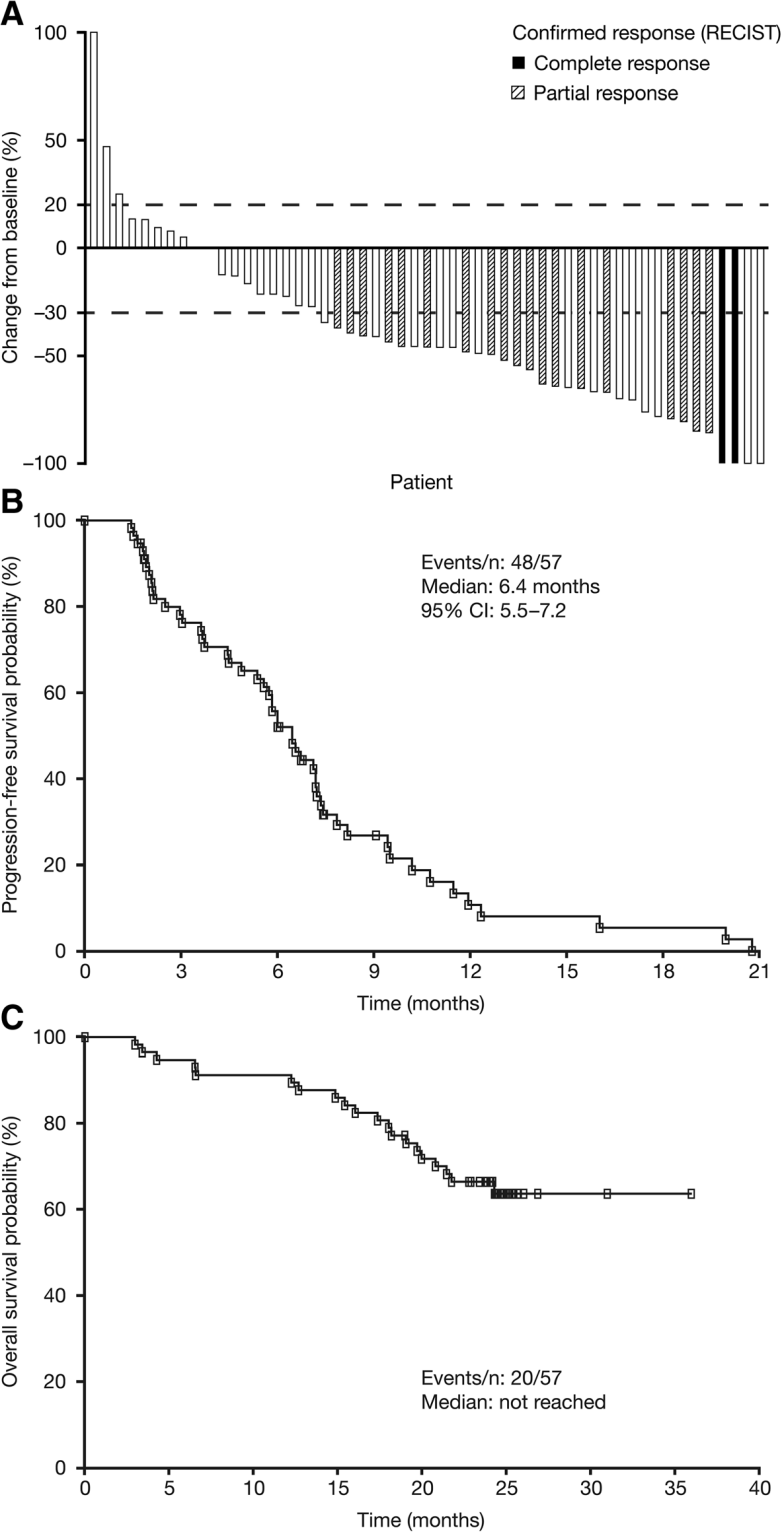
**Antitumor activity of sunitinib plus trastuzumab. (A)** Maximum reduction in target lesion size by patient, with confirmed responses based on RECIST indicated. Broken gray lines indicate cut-offs for progressive disease and partial responses. **(B)** and **(C)** Kaplan–Meier estimates of **(B)** progression-free survival and **(C)** overall survival. RECIST, Response Evaluation Criteria in Solid Tumors.

The confirmed ORR in the efficacy-evaluable population was 37%, with a CBR of 56% (Table 2). The median duration of response was 5.9 months (95% CI: 5.2–7.6). The majority of confirmed responses (71%; 15/21 patients) were reported among patients who were treatment-naïve or had received only adjuvant therapy. For this group, the ORR was 44% and the CBR was 59%. The ORR was numerically higher among patients with visceral versus non-visceral disease (44% vs. 19%, respectively) and among those with estrogen-receptor-negative versus -positive disease (41% vs. 33%). Overall, the majority of patients (43/57 evaluable patients, 75%) had reductions in tumor size over the course of the study (Figure 1A).

**Table 2:** Summary of tumor response with sunitinib plus trastuzumab

	**n (%)**						
**Response parameter**	**All patients**	Treatment-naïve or prior adjuvant treatment* only	Prior first-line treatment*	**Visceral disease**^**†**^	**Non-visceral disease**	**Estrogen-receptor positive**	**Estrogen-receptor negative**
	**(n = 57)**	**(n = 34)**	**(n = 23)**	**(n = 41)**	**(n = 16)**	**(n = 30)**	**(n = 27)**
Complete response	2 (4)	2 (6)	0	1 (2)	1 (6)	2 (7)	0
Partial response	19 (33)	13 (38)	6 (26)	17 (41)	2 (13)	8 (27)	11 (41)
Stable disease	21 (37)	10 (29)	11 (48)	15 (37)	6 (38)	13 (43)	8 (30)
24 weeks	11 (19)	5 (15)	6 (26)	6 (15)	5 (31)	6 (20)	5 (19)
Objective response	21 (37)	15 (44)	6 (26)	18 (44)	3 (19)	10 (33)	11 (41)
95% exact CI	24–51	27–62	10–48	28–60	4–46	17–53	22–61
Clinical benefit^‡^	32 (56)	20 (59)	12 (52)	24 (59)	8 (50)	16 (53)	16 (59)
95% exact CI	42–69	41–75	31–73	42–74	25–75	34–72	39–78

*Chemotherapy, trastuzumab, and/or lapatinib.

^†^Liver and/or lung.

^‡^Objective response or stable disease ≥ 24 weeks.

At a median duration of follow-up of 24.4 months (95% CI: 24.2–24.9), median PFS was 6.4 months (Figure 1B). Median OS had not yet been reached (Figure 1C); the 1-year survival rate was 91% (95% CI: 80–96%).

### Safety

The most commonly reported non-hematologic AEs of any cause were fatigue/asthenia (75%), diarrhea (60%), and stomatitis/related oral disorders (53%; Table 3). The most common non-hematologic grade 3 AEs were fatigue/asthenia (20%), hypertension (13%), and decreased appetite (7%). There were six non-hematologic grade 4 AEs (LVEF decline, pulmonary embolism, hyponatremia, multi-organ failure, aspartate aminotransferase increase, and pancreatitis). One patient died on study from cardiogenic shock; prior to enrolling in the present study, this patient had received a combination of fluorouracil, cyclophosphamide, and epirubicin in the adjuvant setting and trastuzumab followed by lapatinib in the advanced/metastatic setting.

**Table 3:** Non-hematologic adverse events (AEs) and hematologic laboratory abnormalities (N = 60)

**AE or laboratory abnormality**	**n (%)**			
	**Grade 1/2**	**Grade 3**	**Grade 4**	**Any grade**
Non-hematologic AEs of any cause occurring in ≥ 15% of patients				
Fatigue/asthenia	33 (55)	12 (20)	0	45 (75)
Diarrhea	33 (55)	3 (5)	0	36 (60)
Stomatitis, oral discomfort, and related oral syndromes	29 (48)	3 (5)	0	32 (53)
Dysgeusia	27 (45)	0	0	27 (45)
Hypertension	18 (30)	8 (13)	0	26 (43)
Skin and subcutaneous tissue disorders	25 (42)	0	0	25 (42)
Vomiting	19 (32)	2 (3)	0	21 (35)
Dyspepsia	20 (33)	0	0	20 (33)
Epistaxis	18 (30)	2 (3)	0	20 (33)
Nausea	20 (33)	0	0	20 (33)
Headache	17 (28)	2 (3)	0	19 (32)
Decreased appetite	14 (23)	4 (7)	0	18 (30)
Dry skin	14 (23)	1 (2)	0	15 (25)
Ejection fraction decreased	10 (17)	3 (5)	1 (2)	14 (23)
Abdominal pain	11 (18)	0	0	11 (18)
Hand–foot syndrome and related disorders	8 (13)	3 (5)	0	11 (18)
Pyrexia	11 (18)	0	0	11 (18)
Weight decreased	11 (18)	0	0	11 (18)
Constipation	10 (17)	0	0	10 (17)
Left ventricular dysfunction	8 (13)	2 (3)	0	10 (17)
Insomnia	8 (13)	1 (2)	0	9 (15)
Dyspnea	8 (13)	1 (2)	0	9 (15)
Hematologic laboratory abnormalities				
Leukopenia	46 (77)	4 (7)	1 (2)	51 (85)
Anemia	46 (77)	2 (3)	0	48 (80)
Neutropenia	36 (60)	9 (15)	1 (2)	46 (77)
Lymphopenia	36 (60)	6 (10)	1 (2)	43 (72)
Thrombocytopenia	34 (57)	6 (10)	3 (5)	43 (72)

Eleven patients discontinued the study and 18 patients permanently discontinued treatment with one or both study drugs due to AEs considered treatment-related. AEs resulted in temporary treatment discontinuations and/or dose reductions of one or both study drugs in 48 patients.

AEs related to measured LVEF declines were reported in 24 patients (40%) during the study (Table 4). However, these were asymptomatic (CTCAE grade 1/2) in 18 patients (30%); only 10% of patients exhibited symptoms related to LVEF declines. LVEF decline occurred more frequently in patients who had received prior treatment with anthracyclines alone or combined with trastuzumab (55% and 50%, respectively) compared with patients who had received neither type of agent or trastuzumab only (26% and 0%). Median LVEF for the whole group was 63% at baseline; during C3, C5, C7, and C9, and at the end of treatment, it was at or above the lower limit of normal (range: 55–60%).

**Table 4:** LVEF decline* by prior treatment

		**n (%)**		
**Prior treatment**^**†**^	**n**	**Asymptomatic**^**‡**^	**Symptomatic**^**‡**^	**Total**
None	19	5 (26)	0	5 (26)
Trastzumab only	4	0	0	0
Anthracycline only	11	5 (45)	1 (9)	6 (55)
Trastuzumab and anthracycline	26	8 (31)	5 (19)^§^	13 (50)
All patients	60	18 (30)	6 (10)^¶^	24 (40)

*Abbreviations*: *AE* adverse event, *CTCAE* Common Terminology Criteria for Adverse Events, *LVEF* left ventricular ejection fraction.

*Reported as an AE.

^†^Trastuzumab and/or anthracycline.

^‡^Asymptomatic, CTCAE grade 1/2; symptomatic, CTCAE grade 3/4.

^§^One fatal event occurred (cardiogenic shock).

^¶^Three of six of these patients discontinued the study due to LVEF decline.

### Pharmacokinetics

Mean dose-corrected, steady-state trough plasma concentrations (coefficient of variation) of sunitinib, the active metabolite SU12662, and total drug (sunitinib plus SU12662) were 53.5 (52%), 26.7 (54%), and 80.2 (50%) ng/mL on C3D1 (n = 18); and 55.0 (45%), 24.7 (51%), and 79.7 (46%) ng/mL on C5D1 (n = 13), respectively.

### Effect of total-drug exposure on efficacy and safety

Among patients with trough plasma drug concentration measurements on C3D1 or C5D1, the ORR was higher in patients with total-drug trough concentrations above the median (higher-exposure sub-group) than below the median (lower-exposure sub-group; Table 5). SD rates were lower in the former than the latter PK sub-group. CBRs (defined in these analyses as percentages of patients with objective responses or SD ≥ 12 weeks) were similar in the two PK sub-groups. Median PFS was longer in the sub-group with higher exposure on C5D1 (p = 0.013; Table 5).

**Table 5:** Effect of total-drug* exposure on antitumor activity

	**C3D1**		**C5D1**	
**Efficacy parameter**^**†**^	**No. of evaluable patients**	**No. with parameter (%)**	**No. of evaluable patients**	**No. with parameter (%)**
Objective response				
< Median trough concentation	14	4 (29)	9	3 (33)
≥ Median trough concentation	15	10 (67)	9	7 (78)
Stable disease				
< Median trough concentation	14	8 (57)	9	6 (67)
≥ Median trough concentation	15	3 (20)	9	2 (22)
Clinical benefit^‡^				
< Median trough concentation	14	11 (79)	9	9 (100)
≥ Median trough concentation	15	13 (87)	9	9 (100)
Median PFS (95% CI), months				
< Median trough concentation	14	6.6 (4.9–9.5)	9	7.2 (6.0–7.3)
≥ Median trough concentation	15	7.3 (6.4–10.2)^§^	9	9.5 (8.1–10.7)^¶^

Abbreviations: *C* cycle, *D* day, *PFS* progression-free survival.

*Sunitinib + SU12662.

^†^By trough concentration of total drug on C3D1 or C5D1 as indicated.

^‡^Patients with objective responses or stable disease ≥ 12 weeks.

^§^Log-rank p = 0.879.

^¶^Log-rank p = 0.013.

The effect of total-drug trough concentrations on C3D1 (n = 29) and C5D1 (n = 18) on the incidences of specific AEs reported in C1–C4 was also evaluated. Incidences of asthenia (any grade) and leukopenia (any grade and grade 3/4) appeared to be similar or higher in the higher-exposure sub-group. Incidences of any grade and grade 3/4 hypertension and thrombocytopenia appeared to be consistently higher in the higher-exposure sub-group. No lymphopenia was reported in either sub-group, and no consistent trends were observed with respect to the incidences of decreased ejection fraction or neutropenia reported as AEs. Correlative analyses showed moderate to strong correlations between total-drug trough concentrations and a number of key safety parameters (reduced neutrophil and leukocyte counts, elevated systolic or diastolic blood pressure, and reduced LVEF), particularly on C5D1 (Table 6).

**Table 6:** Correlation between trough concentration of total drug* and change in laboratory parameters

**Laboratory parameter**^**†**^	**Pearson correlation coefficient (R)**^**‡**^	
	**C3D1 (*n*** **= 29)**	**C5D1 (*n*** **= 18)**
Absolute neutrophil count	0.15	-0.64
Leukocyte count	0.20	-0.79
Lymphocyte count	0.12	-0.21
Thrombocyte count	-0.33	-0.40
Systolic blood pressure	0.18	0.68
Diastolic blood pressure	0.06	0.51
Ejection fraction	-0.61	-0.21

*Abbreviations*: *C* cycle, *D* day.

*Sunitinib + SU12662.

^†^Based on percent change from baseline.

^‡^Weak correlation, |R| <0.50; moderate correlation, 0.50 ≤ |R| < 0.75; strong correlation, |R| ≥ 0.75.

### Patient-reported outcomes

Mean changes from baseline in EORTC QLQ-C30 functional and symptom scores and in BR23 scores were analyzed. Overall, PROs appeared to be mixed in the study, with some functional domains and symptoms improving during treatment and others (particularly diarrhea) worsening.

Among EORTC QLQ-C30 functional scores, emotional function showed clinically meaningful improvement on C5D1 and C7D1. Role functioning and global health status exhibited clinical meaningful worsening on C3D1 and C5D1. Among symptom scores, pain (C3D1 and C5D1) and insomnia (C5D1 and C7D1) showed improvement. Worsening was observed in fatigue (C3D1) and diarrhea (C3D1, C5D1, and C7D1). Among BR23 scores, improvement was observed in breast symptoms (C3D1, C5D1, and C7D1) and arm symptoms (C5D1). Sexual enjoyment (among those who reported being sexually active) and systemic therapy side effects worsened by C3D1. No other clinically meaningful changes in EORTC QLQ-C30 or BR23 scores were observed.

## Discussion

Sunitinib 37.5 mg on a CDD schedule in combination with trastuzumab (weekly or 3-weekly) demonstrated substantial antitumor activity in patients with ABC, with an ORR of 37% and a CBR of 56%. The null hypothesis that the ORR of the sunitinib - trastuzumab combination was no different than the historical trastuzumab ORR was therefore rejected. A 1-year survival rate of 91% was achieved, and median OS was not reached (survival was only followed for 2 years post-dose).

The ORR of the combination was greater than the 11% ORR reported in two previous studies of sunitinib monotherapy (administered at 50 mg/day on Schedule 4/2 or 37.5 mg/day on a CDD schedule) in previously treated patients with ABC [13,32]. In the current study, most responses (71%) were noted in treatment-naïve patients and in patients who had received only prior adjuvant therapy. These patients achieved an ORR of 44%, similar to that observed in an earlier trial of first-line bevacizumab plus trastuzumab (46%) [21]. The high ORR obtained with the sunitinib–trastuzumab combination in patients with visceral disease (44%) was also encouraging. These observations provide additional support for synergy between tumor-specific HER2-targeted and antiangiogenic therapies for aggressive disease, as predicted in preclinical studies (Pfizer Inc., data on file). In particular, the action of sunitinib on both vascular endothelial cells and pericytes [7,8,33] as a result of dual targeting of VEGFR and PDGFR may complement HER2-targeting by trastuzumab, although trastuzumab resistance did not appear to be overcome in patients receiving the combination as second-line therapy.

In general, the sunitinib - trastuzumab combination appeared to have an acceptable safety profile that was broadly consistent with the profiles of both drugs administered as monotherapy [13,32,34], with the majority of AEs being of mild to moderate severity. Dosing modifications were frequently used to manage AEs, with 80% of patients having temporary treatment discontinuations and/or dose reductions of one or both study drugs due to AEs.

Cardiac dysfunction is a known side effect of trastuzumab, with reported incidences of 3–7% when given as monotherapy and 27% when administered with anthracycline-containing chemotherapy [35]. Cardiac dysfunction has also been associated with sunitinib treatment, with reported incidences of 11–19% in patients with RCC or GIST [36-38]. Given that the drugs were used in combination in this study, LVEF was monitored frequently. Forty percent of patients (24/60) experienced AEs related to measured LVEF declines, although in the majority of these patients (75%) the events were asymptomatic (CTCAE grade 1/2), with measured changes in LVEF during the events being between -4% and -37% relative to baseline. Pre-exposure to anthracyclines appeared to be a major factor contributing to cardiac dysfunction: 19 of the 24 patients with LVEF-related AEs overall (79%) and all six with symptomatic LVEF-related events had received prior anthracycline treatment, either with or without prior trastuzumab therapy. Of the 23 patients in the study who had not received prior anthracyclines, only five experienced LVEF-related AEs (22%). This rate of LVEF decline following prior anthracycline treatment was consistent with that reported in other studies using trastuzumab [2,22]. Median baseline LVEF was similar between the 37 patients who had received prior anthracyclines and the 23 who had not (61% vs. 64%).

In total, five patients (8%) discontinued the present study for reasons related to cardiac dysfunction: three discontinued due to measured LVEF declines (ranging from -18% to -30% relative to baseline), one due to acute heart failure (with an LVEF decline of 27%), and one due to asthenia, cardiac insufficiency, and dyspnea (with an LVEF decline of 37%). Additionally, as noted above, one patient died due to cardiogenic shock (with a change in LVEF from 54% at screening to 23% 3 days prior to death). In the majority of the 24 patients with LVEF-related AEs (67%), the events resolved either spontaneously or following temporary or permanent discontinuation of trastuzumab (and, in one case, sunitinib as well), in contrast to cardiac dysfunction associated with anthracycline treatment, which is usually irreversible [39]. Nevertheless, the results of the present study, including the high rate of grade 3/4 cardiotoxicity in anthracycline-exposed patients, indicate that great caution along with proactive cardiac monitoring and management of cardiotoxicity by treatment interruption/discontinuation are critical when using a drug combination such as that tested in this study.

Steady-state concentrations of sunitinib, the active metabolite SU12662, and total drug were consistent with those obtained with single-agent sunitinib administered at 50 mg/day on Schedule 4/2 to patients with MBC [13]. Pharmacokinetic evaluations suggested that no clinically relevant drug - drug interactions had occurred. In addition, antitumor response appeared to correlate with plasma drug exposure: ORR and PFS appeared to be greater in patients with higher plasma drug exposures. Correlations were also observed between plasma drug exposures and several key safety parameters.

In conclusion, sunitinib on a CDD schedule in combination with trastuzumab (weekly or 3-weekly) demonstrated antitumor activity in patients with HER2-positive ABC, particularly in those who were treatment-naïve or had only received prior adjuvant treatment. Sunitinib plus trastuzumab had acceptable safety and tolerability in patients with ABC who had not received prior anthracycline therapy. The regimen is not being developed further, however, based on disappointing results obtained in four phase III studies of sunitinib in patients with ABC [26-29]. Nevertheless, the results obtained in this study contribute to the field of antiangiogenesis by adding to the evidence supporting a beneficial effect of targeting both the VEGF and HER2 pathways and by providing a platform for further exploration with other agents that may lead to benefit in specific patient populations.

## Competing interests

This study was sponsored by Pfizer Inc. T. Bachelot has had consultant/advisory relationships with and received honoraria from Novartis and Roche and has received research funding from Roche. S. Verma has had consultant/advisory relationships with and received honoraria from Roche and Pfizer, and has received research funding from Roche. X. Pivot has had consultant/advisory relationships with Roche, GlaxoSmithKline, and Novartis, and received honoraria from Roche and GlaxoSmithKline. M. F. Kozloff has received honoraria from Pfizer and Genentech. C. Prady has received honoraria from Roche. X. Huang, R. Khosravan, Z. Wang, V. Tassell, and K. A. Kern are/were employees of Pfizer and hold/held Pfizer stock. R. Cesari is an employee of Pfizer. J.-Y. Blay has had consultant/advisory relationships with and received honoraria from Pfizer, Novartis, Roche, GlaxoSmithKline, and Pharmamar, and has received research funding from Novartis, Roche, and Pharmamar. The other authors have no potential conflicts of interest to disclose.

## Authors’ contributions

J-YB contributed to the conception and design of the study. TB, JAG-S, SV, MG, CP, and AL participated in collection and assembly of the data. TB, XP, ZW, MFK, CP, XH, RK, RC, VT, KAK, and J-YB participated in data analysis and interpretation. All authors participated in drafting the manuscript and/or revising it critically for important intellectual content, and all approved the final version.

## Pre-publication history

The pre-publication history for this paper can be accessed here:

http://www.biomedcentral.com/1471-2407/14/166/prepub
